# Herpes Simplex Virus Esophagitis as a Presentation of Febrile Neutropenia: A Case Report

**DOI:** 10.7759/cureus.31280

**Published:** 2022-11-09

**Authors:** Sammy Droubi, Pinang Shastri, Nicholas Yared, Dalia Ibrahim, Hend Elsaghir

**Affiliations:** 1 Radiology, University of Toledo Medical Center, Toledo, USA; 2 Internal Medicine, University of Toledo Medical Center, Toledo, USA; 3 Infectious Diseases, Henry Ford Health System, Detroit, USA; 4 Pathology, University of Toledo Medical Center, Toledo, USA; 5 Infectious Diseases, University of Toledo Medical Center, Toledo, USA

**Keywords:** immunocompromised patient, esophageal ulcerations, opportunistic infection, febrile neutropenia, herpes simplex virus esophagitis

## Abstract

Herpes Simplex Virus esophagitis typically manifests as mucocutaneous lesions in immunocompromised patients, most frequently in organ and bone marrow transplant recipients. However, it has not been appropriately reported as a cause of febrile neutropenia despite being a relatively common opportunistic infection in this patient population.

A 58-year-old man recently diagnosed with Ewing Sarcoma for which he was receiving chemotherapy presented with febrile neutropenia. Following a prolonged hospital course characterized by persistent fevers, an endoscopic evaluation was performed and diagnosis of Herpes Simplex Virus esophagitis was confirmed via histopathology. Prompt administration of acyclovir resulted in the complete resolution of the patient’s symptoms.

Recognition of Herpes Simplex Virus esophagitis as an etiology of febrile neutropenia can ensure more prompt diagnosis and allow for appropriate management of these patients. In addition, this case report emphasizes a need for further research into additional diagnostic markers in the workup of these patients and the incorporation of antiviral therapy in febrile neutropenia algorithms.

## Introduction

Febrile neutropenia is a common presentation requiring a broad differential to properly identify its cause and targeted therapy. This differential includes bacterial or viral pneumonia, urinary tract infection, port infection, and invasive fungal infection among other etiologies. Identifying the causes of febrile neutropenia can decrease associated healthcare costs, unnecessary and invasive testing, and most importantly, patient morbidity and mortality [1]. One such presentation of febrile neutropenia that is notably under-reported in the medical literature is Herpes Simplex Virus (HSV) esophagitis. A relatively common culprit in the immunosuppressed patient population, HSV esophagitis is still easily overlooked due to its ambiguous symptoms and lack of documented association with this condition. Also, as antivirals are not typically used early in the course of febrile neutropenia, delayed or missed diagnosis of HSV esophagitis can lead to mismanagement of these patients and increased length of hospital stay. Therefore, increased recognition of this etiology of febrile neutropenia is paramount to effectively managing this often challenging patient presentation. We present here a case in which a patient admitted to the hospital with febrile neutropenia was subjected to an increased length of stay, delayed diagnosis, and delayed treatment. This was due in part to the lack of awareness of HSV esophagitis as a potential cause of febrile neutropenia caused by the scarcity of this association in the medical literature.

## Case presentation

A 58-year-old man diagnosed with Ewing Sarcoma of the left lower extremity 6 weeks earlier, receiving chemotherapy with vincristine, doxorubicin, and cyclophosphamide alternating with ifosfamide and etoposide (VAC-IE), began experiencing general malaise and weakness for two days. This was associated with a sore throat and a mildly productive cough. He subsequently called his oncologist who recommended hospital admission.

Vital signs included a fever of 102.9º F, heart rate of 119, respiratory rate of 18, and blood pressure of 108/62 mmHg. Physical examination revealed an ill-appearing man with warm, clammy skin and white plaques noted along his oropharynx and tongue. His initial labs were significant for neutropenia. The patient was identified as a presumed case of febrile neutropenia. His initial workup included a chest X-ray, blood cultures, sputum culture, and a urine culture. Chest X-ray was clear and revealed no abnormality. Serial blood cultures yielded no growth. Urine culture also demonstrated no growth. Sputum culture resulted in light growth of colonies consistent with upper respiratory flora.

He was empirically started on IV cefepime 2 grams every 8 hours and vancomycin 750 m every 6 hours, as well as oral fluconazole 100 mg once a day due to concern for candidal esophagitis. Five days into hospitalization, his fever persisted with temperature readings at around 103º F, and he became increasingly pancytopenic (Table 1).

**Table 1:** Cell count.

	Admission	5 days into hospitalization	9 days into hospitalization	
White blood cells (WBC) (10^3^/uL)	0.84	0.63	3.47	Normal: 4.00-10.60
Red blood cells (RBC) (10^6^/uL)	2.43	2.20	2.57	Normal: 4.20-5.70
Platelets (10^3^/uL)	16	9	63	Normal: 150-400
Absolute neutrophils (10^3^/uL)	0.4	0.1	2.4	Normal: 1.6-7.6
Absolute Lymphocytes (10^3^/uL)	0.2	0.0	0.0	Normal: 1.2-4.0

**Figure 1:**
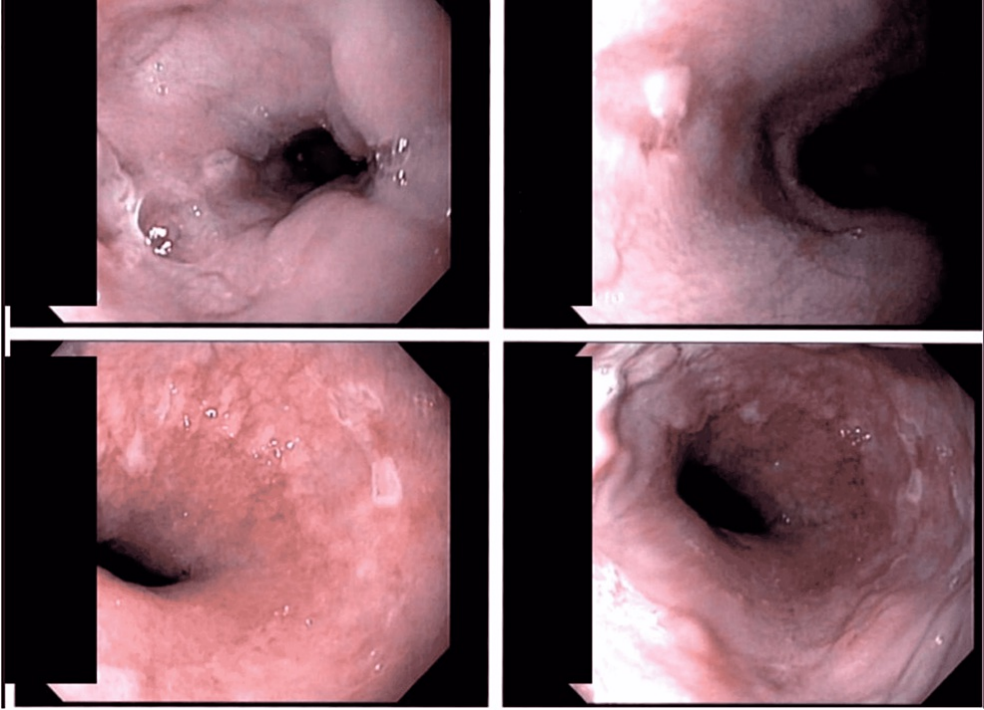
Endoscopic images revealing scattered discrete esophageal ulcerations of varying sizes interspersed with normal esophageal mucosa.

Seven days into hospitalization, the patient complained of worsening odynophagia. He also began to develop blistering along his lip, suggestive of herpes labialis. Nine days into hospitalization, the patient underwent an esophagogastroduodenoscopy, which revealed scattered discrete esophageal ulcerations of varying sizes with normal esophageal mucosa in between, concerning for viral esophagitis (Figure 1).

**Figure 2:**
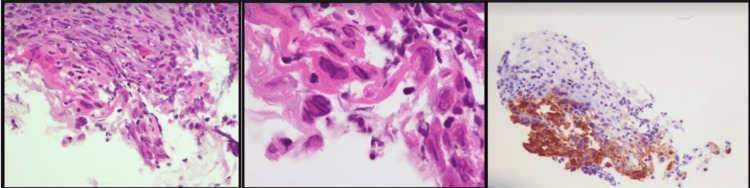
Histopathologic analysis of the biopsied esophageal ulcerative lesions. Histopathologic analysis of the biopsied esophageal ulcerative lesions with low (left) and high (middle) power views of esophageal epithelium demonstrating Herpes Simplex Virus infection of the squamous cells with multinucleation, nuclear molding, chromatin margination, nucleomegaly, and nuclear inclusions. Immunohistochemical staining (right) for Herpes Simplex Virus 1/2 reveals numerous positive inclusions.

Soft tissue biopsy of the esophagus was then obtained. Of note, while the patient’s cell counts were improving nine days into hospitalization, he maintained a fever of 102.4º F, presumably due to his persistent lymphopenia (Table 1). He was started on intravenous (IV) acyclovir 350 mg administered every 8 hours and within one day, his odynophagia and fever began to improve, with his temperature falling to 99.5º F. Pathologic analysis of his esophageal biopsy (Figure 2) confirmed HSV esophagitis as the diagnosis.

The patient was subsequently discharged 10 days into hospitalization and maintained on a prophylactic oral acyclovir regimen of 800 mg twice daily for his remaining eight (VAC-IE) chemotherapy cycles without an episode of recurrence.

## Discussion

We propose that HSV esophagitis be considered a cause of febrile neutropenia. Currently, there is a lack of awareness of this association due to its scarcity in the literature, the non-specific symptoms in this patient population, and an emphasis on the more common causes of febrile neutropenia. HSV infection is believed to result from decreased cell-mediated immunity rather than neutropenia [2]. For this reason, the diagnosis of HSV esophagitis can be overlooked in these febrile neutropenic patients because clinicians may focus on alternate conditions more typically associated with neutropenia. Although a thorough daily physical exam and maintaining a broad differential proved crucial in the identification of HSV esophagitis in our patient, this case emphasizes a need for additional considerations in the diagnostic workup and resultant management of these patients.

While the association between HSV esophagitis and febrile neutropenia has been mentioned [2, 3], it is surprisingly scarce in the medical literature. Approximately two-thirds of high-risk neutropenic patients develop HSV reactivation [4], and HSV esophagitis causes fever in about half of patients [5, 6]. Thus, there is a high probability that certain neutropenic patients could develop HSV esophagitis and present with febrile neutropenia. However, there continues to be a diminutive role of HSV within many guidelines regarding the workup and management of febrile neutropenic patients [7-11].

HSV esophagitis is most often caused by the reactivation of HSV type 1 in immunocompromised patients. Symptoms are often vague in this patient population. These include odynophagia, dysphagia, retrosternal chest pain, and fever in approximately 50% of patients. [5, 6] While our patient did endorse some of these symptoms, they were initially presumed to be due to thrush or candidal esophagitis, which are considerably more common. In addition, the patient at that time had white plaques that characterize candidiasis in his mouth on exam and there were no overt signs of a viral etiology. Later on, blistering of the patient’s lip prompted evaluation for HSV esophagitis, and the proper workup was initiated to confirm the diagnosis. Following 10 days of persistent fevers, initiation of IV acyclovir 350 mg every 8 hours led to the resolution of fevers within just one day, despite normalization of his absolute neutrophil count several days earlier. The efficacy of IV acyclovir had also previously been demonstrated in a patient population similar to our patient’s by Kubesova et al. [3], further supporting its potential clinical benefit.

A systematic approach to chemotherapy-induced febrile neutropenia involves the use of empiric therapy to cover the most common and concerning etiologies. However, multiple etiologies are often overlooked and undertreated. Antibacterial treatment is typically begun as soon as blood cultures are drawn, and antifungals are generally not used unless the patient’s fever persists past 4-7 days and there is no identified source of infection [8, 11]. Antiviral therapy is currently not indicated unless there is active evidence of a specific viral infection [8]. However as demonstrated in our patient’s case, this may be difficult to determine. Attributing our patient’s mucositis to fungal esophagitis resulted in improper treatment, unnecessary testing, and prolonged hospitalization. Thus, additional considerations in the management of febrile neutropenic patients should focus on more quickly identifying potential non-bacterial sources to help initiate appropriately targeted therapy.

Current recommendations about giving antiviral therapy in the setting of febrile neutropenia are insufficient in cases where HSV esophagitis is suspected given the ambiguity of symptoms in these patients. Additional diagnostic markers should be considered when deciding whether to start antiviral therapy empirically [8]. One proposed diagnostic approach would be to include HSV serology earlier in the workup of a febrile neutropenic patient. Another possibility would be to use HSV serology to establish seropositivity for all chemotherapy patients with a high risk for neutropenia. While seropositivity has been used in the past to determine antiviral prophylaxis in this population, studies have shown conflicting results [7, 8, 9, 12]. We instead propose further investigation of its use as a marker to determine the risk of HSV reactivation in the setting of febrile neutropenia. This incorporation of HSV serology prior to or early in the patient’s course could allow for more prompt identification of this etiology and subsequent treatment. However, there have been documented limitations regarding the sensitivity and specificity of HSV serology [13]. Alternatively, a febrile neutropenia protocol that targets etiologies chronologically in order of their likelihood at designated time intervals could also prove beneficial in ensuring timely diagnosis and treatment. For example, if the patient shows no response to antibiotics after 4-7 days, antifungals are recommended [9]. Including antivirals at a designated time in this protocol could improve patient management. Further research into these potential changes would ensure the most effective course.

## Conclusions

Herpes Simplex Virus esophagitis is an understated cause of febrile neutropenia, most frequently in organ and bone marrow transplant recipients. Recognition of Herpes Simplex Virus esophagitis as a potential etiology of febrile neutropenia can ensure a more prompt and efficient diagnosis. As a result, it would ensure appropriate management of these patients as well. This case report emphasizes a need for further research into additional diagnostic markers in the workup of these patients and the incorporation of antiviral therapy in febrile neutropenia algorithms. This case introduces an underreported presentation of febrile neutropenia, emphasizing the importance of its timely recognition and the need to reconsider the role of additional diagnostic markers and antiviral therapy in febrile neutropenia algorithms. 
